# 16.2 CHILDHOOD TRAUMA ENGAGES OXIDATIVE STRESS, HIPPOCAMPUS ALTERATIONS, AND POORER CLINICAL OUTCOME IN EARLY PSYCHOSIS PATIENTS

**DOI:** 10.1093/schbul/sby014.061

**Published:** 2018-04-01

**Authors:** Luis Alameda, Margot Fournier, Ines Khadimallah, Philippe S Baumann, Martine Cleusix, Alessandra Griffa, Paul Klauser, Raoul Jenni, Michel Cuenod, Patric Hagmann, Philippe Conus, Kim Do

**Affiliations:** 1 Institute of Psychiatry, Psychology & Neuroscience, King’s College London; 2 Center for Psychiatric Neuroscience, Lausanne University Hospital; 3 Lausanne University; 4 Signal Processing Laboratory, Ecole Polytechnique Fédérale de Lausanne, Lausanne University Hospital; 5 The University of Melbourne, Melbourne Neuropsychiatry Centre; 6 Center for Psychiatric Neuroscience, University Hospital of Lausanne - CHUV Cery; 7 Service of General Psychiatry, Lausanne University Hospital

## Abstract

**Background:**

Exposure to childhood trauma (CT) is a global major public-health and social-welfare problem worldwide. CT increases the vulnerability to major psychiatric conditions including psychosis and is associated with poorer clinical outcome. CT affects the development of brain structures such as hippocampus, possibly through oxidative stress and neuroinflammation, two mechanisms linked to psychosis. We therefore hypothesized that there is an interplay between oxidative stress and CT in psychosis patients. We thus explored in early psychosis patients the relationships between CT and i) hippocampal volume, ii) antioxidant systems; and iii) clinical and cognitive outcomes.

**Methods:**

We studied a cohort of 118 early psychosis patients, 36 were exposed to CT (experiences of physical, sexual, or emotional abuse/neglect before16 years old). In a subgroup of 48 patients (18 CT), hippocampal volume was determined by MRI. Antioxidant systems were quantified in blood for the whole cohort. Markers were: glutathione peroxidases (GPx) activity which appeared as a peripheral correlate of brain GSH levels (Xin &al, 2016); peroxiredoxine levels (Prx); Thioredoxine (Trx). Psychopathology (PANSS) and neuropsychology (MCCB) were assessed. The various groups were segregated by linear discriminant analysis.

**Results:**

The previously observed decreased hippocampal volume in patients and association of small hippocampal volume with high blood GPx activity (reflecting high oxidative status) (Baumann &al, 2016) was due to the contribution of the traumatized group. Indeed, this association was absent in the no-trauma group, suggesting that the smaller hippocampus is linked to a redox dysregulation. To explore that point further, four groups were then formed, according to trauma and oxidative status: (i) noCT-lowGPx, (ii) noCT-highGPx, (iii) CT-lowGPx and (iv) CT-highGPx. Group CT-highGPx only had smaller hippocampi.

In CT patients, small hippocampal volume was associated with high GPx activity, while hippocampal volume was similar in CT patients with low GPx activity (CT-lowGPx) and in patients not exposed to CT. Interestingly, other antioxidant defense systems such as Trx and oxidized Prx levels correlated negatively with GPx in CT-lowGPx group, suggesting that the Trx/Prx system is able to compensate for changes/decreases in GPx activity. Moreover, CT-lowGPx patients perform better than the other patients on speed of processing, verbal memory and attention tests.

In contrast, hippocampal volume was decreased in CT patients with high GPx activity (CT-highGPx) compared with CT-lowGPx patients and patients not exposed to CT. There was no correlation between GPx and Trx/Prx system in this group. CT-highGPx patients had more severe positive, negative and disorganized symptoms than the other patients.

**Discussion:**

We report that traumatized psychosis patients with high peripheral oxidation status (high GPx) had smaller hippocampal volumes and more severe clinical symptoms, while those with lower oxidation status (low GPx) had better cognition and appear to activate a compensatory antioxidant regulation by the Trx/Prx system.

These results suggest that, in early psychosis patients, traumatic experiences during childhood interact with different redox systems and have long term neuroanatomical and clinical impacts. Therefore, redox pathways such as GPx, Trx and Prx systems represent important translational biomarkers for patient selection and stratification in order to aid in diagnostics and treatment decision at early stages of the disease.

